# The Irish Nursing Board

**Published:** 1917-09-08

**Authors:** 


### THE IRISH NURSING BOARD.

**To What it owes its Being. Its Bye-laws provide for little else than the forming of a Register. Vital Principles: These are suspended by the provision that "all the Members of the Board, save the Representatives elected by the College of Surgeons, shall serve for six years and then be eligible for Re-election." Democratic Government is thus eliminated from this Board. Such a Rule, if it may pass as Democratic in Ireland, is foredoomed to Rejection by British Nurses.**

Before proceeding to discuss the bye-laws of this Board, it may be well to state briefly exactly what the institution stands for and of what it consists.

Prior to the genesis of the College of Nursing we had in Ireland a society called the Irish Nurses' Association, which consisted of an Executive Committee, unelected and therefore unrepresentative. Nurses joining the Association paid an annual subscription of half a crown. Ladies wishing to serve on the Executive Committee paid a guinea. No general meetings were held, save, I believe, on two or three special occasions to listen to a professional or semi-professional lecture. The entire policy and *raison d'être* of the Association was to further the cause of State Registration under the direction and auspices of the Central Committee in London, and in this the general body of nurses who belonged to the Association passively acquiesced. In a word, the I.N.A. was the Executive Committee; the half-crown subscribers were ciphers. As soon as the College of Nursing appeared on the horizon there was a cleavage, corresponding with the cleavage which existed in Great Britain, and those who decided to hold aloof, and to oppose the College scheme, found themselves forced to some action. In very adroit manner they endeavoured to secure the backing of the Royal Colleges of Physicians and Surgeons to the founding of this Irish Nursing Board. The Physicians rejected the proposal, but the Surgeons, after much button-holing and canvassing, gave it their support. Meanwhile the College of Nursing formed "the College of Nursing Irish Board," representative of all parts of the country. Ulster, being an emphatic supporter, opened offices in Kildare Street, Dublin, and is at present successfully proceeding with propaganda work. This, in a few words, is the situation as it exists.

### The Bye-Laws.

As I pointed out last week, the issue of the bye-laws was accompanied by a circular-letter from Miss Carson Rae, which, I confess, possessed a more lively interest for me at least than the bye-laws, which is of necessity a dry document. Miss Rae openly admits that this is a rival institution with ideals identical with those of the College. She ignores that the College is already established in Ireland with a Board quite as Irish as her own, and she offers no apologia or reason for what journalists would describe as stealing their thunder.

Now as to the bye-laws (which are published *in extenso* with Miss Rae's letter in last week's *Weekly Irish Times*) there is much to be said. But it should be noted at the outset that they contain nothing whatever to indicate that the objects in view correspond with those of the College, and one is therefore forced to the conclusion that Miss Rae's declaration of aim is only a pious hope of her own. The bye-laws provide for nothing else than the forming of a Register, and the conditions necessary for enrolment are elaborately set out. There are to be a Board, an Executive Committee, and a Register—nothing more.

### Vital Principles.

Readers will probably be curious to learn here and now what is the intrinsic difference between the College of Nursing as a rejected institution and this newly constituted Irish Nursing Board, and will wonder what vital principle is at stake which prevents women of talent and intelligence from throwing in their lot with the College. I confess that I am one of those who wonder, especially in view of Miss Carson Rae's confession of faith. And hence my first examination of the bye-laws was with the object of ascertaining by what system the new institution is to be governed. I think I am correct in stating that the question of democratic control was that round which all the controversy centred in Great Britain. Now the scheme of government provided in the bye-laws is sufficiently democratic to satisfy the aspirations of a young Russian. I shall with pleasure set them forth for the information of all whom they may concern, if you so desire. But, having regard to the fact that paper is at present at famine price, I venture to suggest a postponement of their publication until the war is over. For by a subtle clause—after the Prussian rather than the Russian model—it is provided at the outset that "all the members of the Board, save the representatives elected by the College of Surgeons, shall serve for six years, after which they shall be eligible for re-election." I do not think very many people are concerned as to the method of election to be in operation from and after the year 1923. Meanwhile, obviously, members whose names are on the Register other than the Board (if any) will be inarticulate. The appointment of the Executive Committee is equally subtle. "The Executive Committee," runs the bye-law, "shall consist of seven persons, who shall continue in office till the election of a new Executive Committee"—and there is no clue as to when such election is to take place. The intrinsic difference between the two institutions, therefore, so far as control is concerned, would appear to be that the ladies who declined to throw in their lot with the College determined to have an establishment of their own, controlled by themselves for at least six years, after the fashion of the Irish Nurses' Association. And with such an unrepresentative and unelected aristocracy in office they propose to fulfil in Ireland the functions of the College of Nursing. What a lure for the rank and file!

### The Financial Aspect.

This is not to be by any means a cheap institution. The already trained nurse may find shelter within its sacred precincts up to July 1, 1918, on payment of a guinea. In order immediately to attract the wavering it is provided that from July 1, 1918, to July 1, 1919, "all persons who have completed their training before July 1, 1917. . . shall have their names placed on the Register on the payment of four guineas." This, no doubt, will cause a rush. But a hapless fate awaits the probationer now in training, who having paid a substantial fee to her hospital, and worked for a nominal salary for a period of three years, will be admitted to the Board's examination on payment of a fee of three guineas! She will assuredly have earned her diploma when it comes into her possession.

You will have heard it whispered that we love nothing better in Ireland than a faction fight, and likewise, probably, that when English people live long enough in Ireland they become more Irish than the Irish themselves. The establishment of the Irish Nursing Board demonstrates both. The leaders who brought it into being are not Irish.

